# Selective phenol recovery via simultaneous hydrogenation/dealkylation of isopropyl- and isopropenyl-phenols employing an H_2_ generator combined with tandem micro-reactor GC/MS

**DOI:** 10.1038/s41598-018-32269-6

**Published:** 2018-09-18

**Authors:** Shogo Kumagai, Masaki Asakawa, Tomohito Kameda, Yuko Saito, Atsushi Watanabe, Chuichi Watanabe, Norio Teramae, Toshiaki Yoshioka

**Affiliations:** 1 Graduate School of Environmental Studies, Tohoku University, 6-6-07 Aoba, Aramaki-aza, Aoba-ku, Sendai, Miyagi 980-8579 Japan; 2 Frontier Laboratories Ltd., 4-16-20, Saikon, Koriyama, Fukushima 963-8862 Japan; 3 Department of Chemistry, Graduate School of Science, Tohoku University, Aoba-ku, Sendai, Miyagi 980-8578 Japan

## Abstract

The pyrolysis of bisphenol A (BPA), an essential process ingredient used in industry and many everyday life products, helps produce low-industrial-demand chemicals such as isopropenyl- and isopropyl-phenols (IPP and iPrP). In this study, tandem micro-reactor gas chromatography/mass spectrometry combined with an H_2_ generator (H_2_-TR-GC/MS) was employed for the first time to investigate the selective recovery of phenol via simultaneous hydrogenation/dealkylation of IPP and iPrP. After investigating the iPrP dealkylation performances of several zeolites, we obtained full iPrP conversion with over 99% phenol selectivity using the Y-zeolite at 350 °C. In contrast, when applied to IPP, the zeolite acid centres caused IPP polymerisation and subsequent IPP-polymer cracking, resulting in many byproducts and reduced phenol selectivity. This challenge was overcome by the addition of 0.3 wt% Ni on the Y-zeolite (0.3Ni/Y), which enabled the hydrogenation of IPP into iPrP and subsequent dealkylation into phenol (full IPP conversion with 92% phenol selectivity). Moreover, the catalyst deactivation and product distribution over repetitive catalytic use were successfully monitored using the H_2_-TR-GC/MS system. We believe that the findings presented herein could allow the recovery of phenol-rich products from polymeric waste with BPA macro skeleton.

## Introduction

Polycarbonate (PC) is currently the largest consumer of bisphenol A (BPA). Therefore, pyrolysis of PC waste has been widely studied^1–3^ because it allows the recovery of oil and gas from polymeric waste^4,5^. This, by itself, represents a significant advantage to treating polymeric waste combined with other resins and organic additives, which cannot be treated by mechanical recycling and solvolysis^6–13^. However, there are several drawbacks associated with this approach. First, this method generates a mixture of various products like phenol, 4-isopropenylphenol (IPP), 4-isopropylphenol (iPrP) and other alkyl phenols^14–16^, some of which are important chemical feedstock that cannot be properly utilised due to their difficult purification. Secondly, the oil obtained through this approach contains oxygen as part of the phenolic compound, which reduces the calorific value when used as a fuel. Even though IPP and iPrP are the major products in this case, they have very low industrial demand. Moreover, these issues are also observed with other BPA-skeletal polymers such as epoxy resins, polysulfones, bismaleimides, triazines, polyarylates, and BPA-type flame retardants. Nevertheless, the global demand for BPA should increase annually^17^ due to the rapid worldwide growth of automotive, construction, and electrical and electronic application markets. Therefore, it is crucial that new methods are developed for the recovery of useful chemicals from polymeric wastes with BPA skeleton via the pyrolytic approach.

Various researches have investigated catalytic pyrolysis using earth-alkali oxides, hydroxides and zeolites^14–16,18,19^. Among them, Grause *et al*.^18^ achieved the largest BPA yield of 91% at 300 °C in the presence of steam and MgO. However, further decomposition of BPA to phenol, IPP, and iPrP is unavoidable during pyrolysis because of the high temperatures required for this process^14–16,18–20^. Thus, the purity of BPA recovered via pyrolysis of PC waste is too low for it to be used as a process ingredient in the production of resins and flame retardants. Moreover, considering the widespread demand for phenol as a petrochemical feedstock for resins, agrichemicals, and medicinal chemicals, it is more industrially useful to let the low-purity BPA further decompose to phenol than to employ it as obtained.

One challenge that must be overcome to achieve selective phenol recovery from BPA via the pyrolytic approach is the conversion of iPrP and IPP into phenol. In this work, we focused on the use of zeolites as inexpensive heterogeneous shape-selective catalysts capable of participating in gas-solid reactions. This is an important step forward for the field of process technology. The applicability of zeolites to different reactions including alkylation^21–25^, dealkylation^24–30^, and transalkylation^28–35^ of (alkyl)benzenes, such as benzene, toluene, ethylbenzene, xylene, and trimethylbenzenes, has been widely studied. These reactions have been mainly investigated using medium-pore zeolites such as ZSM-5 and MCM-22, as well as large-pore zeolites such as Y-zeolite, *β*-zeolite, mordenite, and faujasites. Apart from the pore size, these types of zeolites also differ in terms of the type and strength of their acid sites. Moreover, Pradhan and Rao^36^ reported that large-pore zeolites such as mordenite, Y-zeolite, and *β*-zeolite allow for the transalkylation of the isopropyl group of diisopropyl benzene, thus yielding benzene.

To the best of our knowledge, the dealkylation of alkyl phenol has only been reported by Verboekend *et al*.^37^, who investigated the dealkylation of *n*-propyl phenol, as a representative of depolymerised lignins and coal, using HZSM-5. Therefore, the knowledge on the dealkylation of alkyl phenols is not well established, and research on the dealkylation of iPrP and IPP remains scant. Furthermore, it is assumed that the direct dealkylation of IPP would be an unfavourable and complicated process because the reactive isopropenyl unit in IPP triggers the polymerisation of the IPP molecules at pyrolytic temperatures, thus enhancing product diversification^20^.

**Figure 1:**
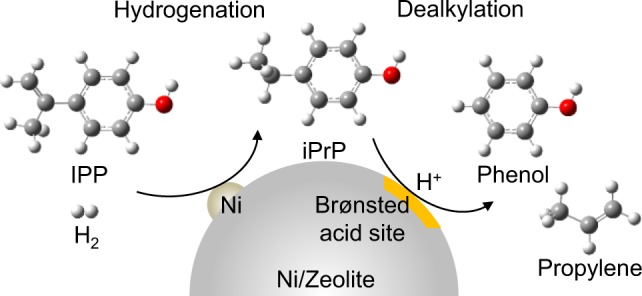
Process of selective phenol recovery from IPP and iPrP using the Ni/zeolite catalyst.

Therefore, we devised a process to selectively recover phenol. This included the hydrogenation of IPP into iPrP and the subsequent dealkylation of iPrP into phenol on Ni-loaded zeolites under H_2_ purge flow (Fig. 1). Ni is known to be a rather inexpensive hydrogenation catalyst of alkyl C=C bonds^38–41^. A number of protocols for the hydrogenation/hydrodeoxygenation procedures of (alkyl)benzenes and phenolic compounds, using Ni-supported catalysts in pressurised batch systems^42–46^, as well as continuous high-pressure H_2_ flow systems and flow systems under atmospheric pressure^47–52^, have been reported. In all these cases, cyclohexane and cyclohexanone are the final products from the deep hydrogenation of aromatic rings or removal of oxygen-containing compounds in product oil by hydrodeoxygenation. Therefore, the selective hydrogenation of the isopropenyl unit and subsequent dealkylation, while preventing the hydrogenation of both the phenol group and the benzene ring on the single catalyst, would be a novel and challenging approach.

**Figure 2:**
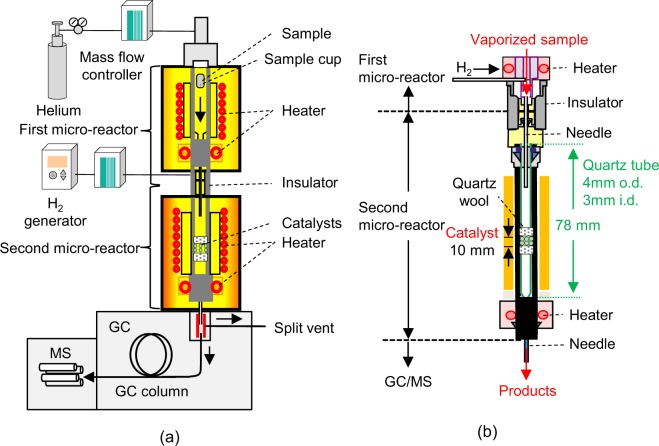
Schemes of the (**a**) H_2_-TR-GC/MS system and (**b**) the second micro-reactor.

In this work, an H_2_-generator combined with tandem micro-reactor (H_2_-TR)-gas chromatography/mass spectrometry (GC/MS), abbreviated to H_2_-TR-GC/MS (Fig. 2) was employed for the first time to reveal the possibility of a selective phenol recovery via simultaneous hydrogenation and dealkylation of iPrP and IPP using Ni-loaded zeolites. Since TR-GC/MS analysis is a relatively new technique, it has mostly been used for simple pyrolysis-catalytic upgrading reactions^53–55^. The first micro-reactor accomodates the pyrolysis or volatilization of the substances and the second micro-reactor is used for catalytic reactions. Researchers have also confirmed the effectiveness of TR-GC/MS analysis in the online monitoring of aromatic hydrocarbon production via the two-step CaO-catalysed pyrolysis of poly(ethylene terephthalate) (PET)^56^ and monitoring of CaO deactivation^57^. The independently controlled furnaces facilitate the evaporation of iPrP and IPP in the first micro-reactor and the reaction with zeolites in the second micro-reactor at their respective optimal temperatures. The products from the TR are directly introduced into the GC/MS system, avoiding a product recovery process and possible product losses by human error, and facilitating rapid catalyst screening.

First, the possibility of iPrP/IPP dealkylation was investigated by screening several zeolites such as ZSM-5 with three different Si/Al ratios, mordenite, Y-zeolite, and ferrierite using TR-GC/MS. Subsequently, Ni-supported Y-zeolites (Ni/Y) with different Ni loadings were synthesised, and their hydrogenation and dealkylation performances toward IPP were evaluated using the H_2_-TR-GC/MS system. Finally, the change in product distribution, caused by the catalyst deactivation due to repeated runs, was monitored using the H_2_-TR-GC/MS system.

## Results and Discussion

### Evaluation of zeolites for the conversion of iPrP into phenol

**Figure 3:**
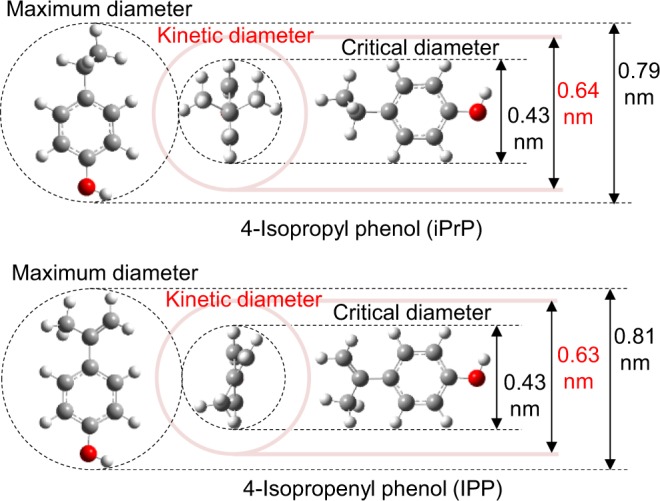
Three-dimensional models and kinetic diameters of the optimised structures of iPrP and IPP.

**Figure 4:**
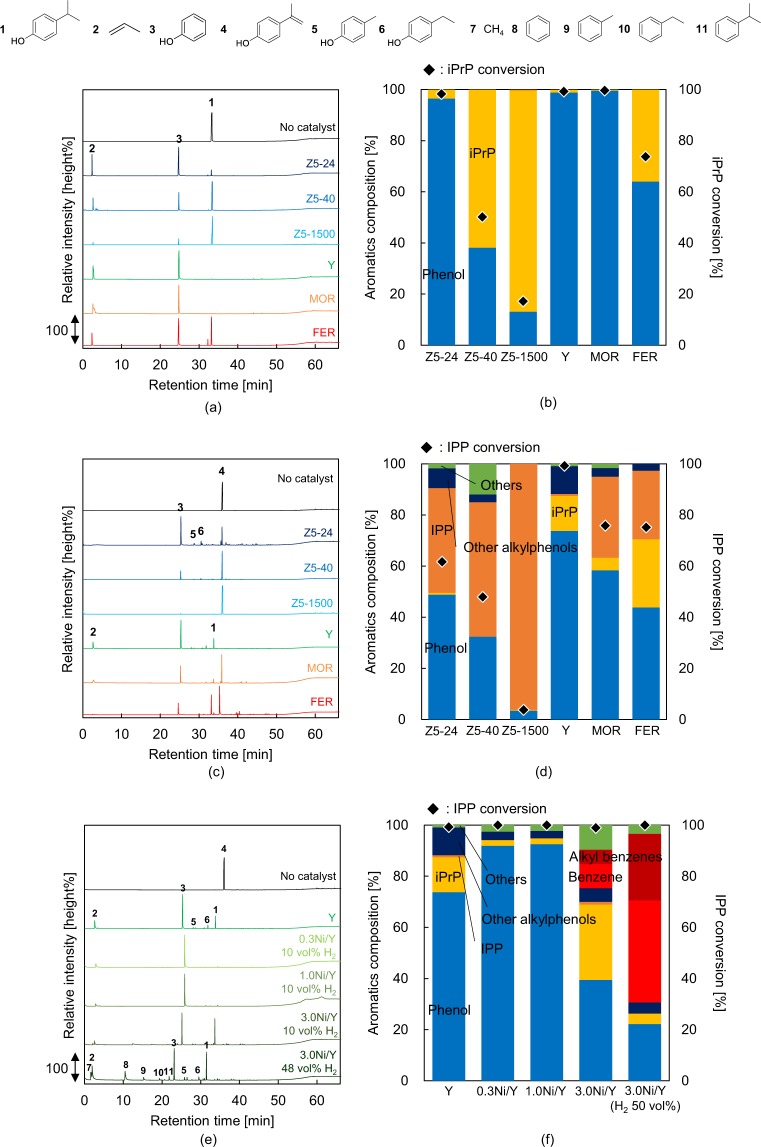
(**a**) GC/MS chromatograms and (**b**) composition of aromatics and iPrP conversion data obtained from the catalytic conversion of iPrP using zeolites. (**c**) GC/MS chromatograms and (**d**) composition of aromatics and IPP conversion data obtained from the catalytic conversion of IPP using zeolites. (**e**) GC/MS chromatograms and (**f**) composition of aromatics and IPP conversion data obtained from the catalytic conversion of IPP using Ni/Y catalysts.

**Figure 5:**
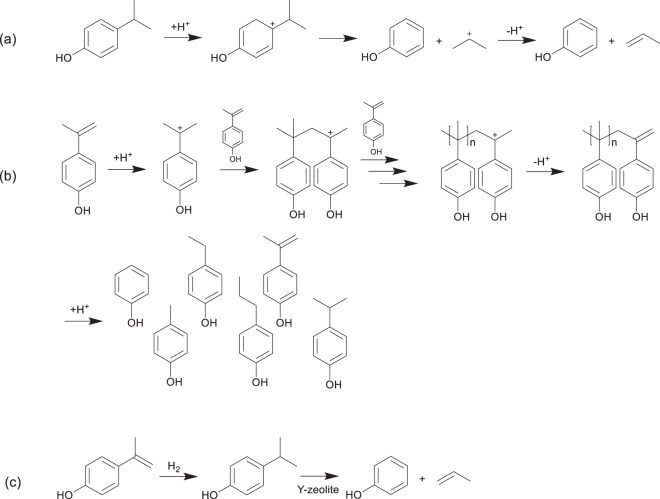
(**a**) Sequence of reactions to the formation of phenol and propylene from iPrP via an acid-catalysed dealkylation of the isopropyl unit. (**b**) Sequence of the acid-catalyzed IPP polymerization and subsequent cracking of the polymerized compounds. (**c**) Overall process of the IPP conversion into phenol via hydrogenation and subsequent dealkylation using the Ni/Y catalysts.

The effects of zeolite properties such as pore size and acid density on the dealkylation of iPrP were investigated using TR-GC/MS. The zeolite properties and abbreviations are summarized in Table 1. Structure optimisation of iPrP and IPP was carried out with Gaussian R 16W^58^ using the density functional theory. The kinetic diameter of these molecules was calculated by the method reported by Wang and Frenklach^59^ (Fig. 3 and SI). Figure 4(a,b) present the GC/MS chromatograms obtained in the presence and absence of each zeolite, as well as the iPrP conversion and aromatics composition data for each catalytic reaction. The chromatographic data show that iPrP was thermally stable under the conditions used herein, and in the absence of zeolite, only iPrP was present (Fig. 4(a)). Moreover, Z5-24 effectively converted iPrP into propylene and phenol (2 and 3, respectively, Fig. 4(a)) with 98% iPrP conversion rate and 96% phenol selectivity (Fig. 4(b)) and without any side reactions. The detailed product distributions are summarised in Table S1 (SI). In contrast, the iPrP conversion rate in the presence of Z5-40 and Z5-1500 catalysts drastically decreased (50% and 17%, respectively). Plots of *C*_iPrP_ *vs*. acid site density of the ZSM-5 zeolites show perfect linearity (Fig. S4, SI). These results revealed that the dealkylation of iPrP progressed on the acid sites, which worked as Brønsted acids (Fig. 5(a)).

**Table 1:** Characteristics of the zeolites used in the present work.

Abbreviated name	Zeolite type	Framework type code	SiO_2_/Al_2_O_3_ ratio^a^ [mol/mol]	Pore diameter^a^ [nm]	Acid site density^b^ [mmol·g^−1^]
Z5-24	ZSM-5	MFI	24	0.58	1.14
Z5-40	ZSM-5	MFI	40	0.58	0.51
Z5-1500	ZSM-5	MFI	1,500	0.58	0.08
Y-zeolite	Y-zeolite	FAU	15	0.90	0.14
MOR	Mordenite	MOR	18	0.70	0.49
FER	Ferrierite	FER	18	0.48	1.45

^a^Details were provided from Tosoh Corporation. ^b^Determined using the area of the NH_3_ temperature-programmed desorption (NH_3_-TPD) profile. NH_3_-TPD profile is summarised in Fig. S1 in the Supporting Information.

Surprisingly, Y-zeolite and MOR achieved almost complete iPrP conversions, although they have lower acid site densities than Z5-40. However, these zeolites have bigger pore sizes than the ZSM-5 zeolites. These trends suggest that bigger pores than the kinetic diameter of iPrP (0.64 nm) are required for effective dealkylation. It has previously reported that the acid sites inside the pores have a significant role in the dealkylation, transalkylation and isomerisation reactions of diisopropyl-benzenes and –naphthalenes^36,60,61^. The high iPrP conversion and excellent phenol selectivity in the presence of Z5-24, which has slightly smaller pores than the kinetic diameter of iPrP, might be due to a dealkylation enhancement caused by the acid sites on the pore entrances^62^. In fact, FER, the zeolite with the smallest pores among all the zeolites studied herein, showed only 74% iPrP conversion despite having the highest acid site density among the tested zeolites. Thus, it can be concluded that dealkylation occurs mainly at the acid sites inside the pores.

### Evaluation of zeolites for the conversion of IPP into phenol

The IPP conversion in the presence and absence of the zeolite catalysts was investigated using TR-GC/MS via the same procedure as for the iPrP conversion. The obtained GC/MS chromatograms, IPP conversion rates, and aromatics composition data are summarised in Fig. 4(c,d). The results confirmed that the bulk of IPP was rapidly evaporated in the first micro-reactor and carried into the second micro-reactor, while trace amounts of phenol, iPrP, other alkyl phenols and IPP dimers were also observed in the absence of a catalyst (Table S2). This suggests that a small-scale IPP pyrolysis reaction^20^ occurred in the reactor. Moreover, the IPP conversion rate and phenol selectivity in the presence of Z5-24 were only 62% and 58%, respectively, much lower than the values obtained for the iPrP conversion. Although the IPP conversion and phenol selectivity decreased with decreasing acid site density of the ZSM-5 zeolites, the IPP conversion was not proportional to the acid site density (Fig. S4). FER, the catalyst with the highest acid site density and smallest pore size, showed higher IPP conversion rate (75%) and a lower phenol selectivity (44%) than the Z5 catalysts. Moreover, propylene was not detected in the presence of ZSM-5 and FER zeolites. These results suggested that the phenol in this reaction was not obtained from a direct IPP dealkylation, but from thermal decomposition of the IPP pyrolysates.

The kinetic diameter of IPP (0.63 nm) is comparable with that of iPrP, suggesting that IPP mainly reacted in the acid sites on the surface of ZSM-5 and FER. In fact, Brzozowski and Skupiński^60,62^ suggested that the external surfaces and pore entrances were also important reaction sites, though the molecules do not enter inside the pores. Moreover, various alkyl phenols were observed in the reactions examined herein in the presence of Z5-24 and Z5-40, while the very weak acid Z5-1500 did not enhance the IPP reaction. This suggest that the acid sites catalysed the IPP polymerisation and subsequently enhanced the cracking of the IPP polymers, resulting in diversification of the alkyl phenols (Fig. 5(b)).

In the presence of MOR, relatively higher IPP conversion (76%) and phenol selectivity (58%) were observed. Moreover, propylene was formed simultaneously with the phenol production. This suggests that IPP could enter the pores, where it transformed into iPrP, which subsequently dealkylated to form phenol (Fig. 5(a)). Both IPP conversion and phenol selectivity increased (over 99% and 74%, respectively) in the presence of the Y-zeolite, which has the largest pores (0.90 nm), thereby favouring the entrance of IPP molecules. The size limitations of the pores could arguably explain the different behaviours of the IPP molecules depending on the pore size. For ZSM-5 zeolites and FER, the IPP molecules mainly reacted at the acidic sites on the surface, which had no space limitations, thereby enhancing the IPP polymerisation. In contrast, the IPP molecules which entered into the pores of the Y-zeolite could not be enlarged by polymerisation due to the limited space inside the pores. According to the optimised structures and kinetic-diameter calculations (Fig. S3, SI), IPP dimers (kinetic diameter: 0.80 nm) were the largest oligomers that could enter the pores, with IPP trimers already being too large. Therefore, the Y-zeolite favoured the decomposition of IPP and IPP dimers into phenol rather than the polymerisation.

### Characterisation of the synthesised Ni/Y catalysts

**Figure 6:**
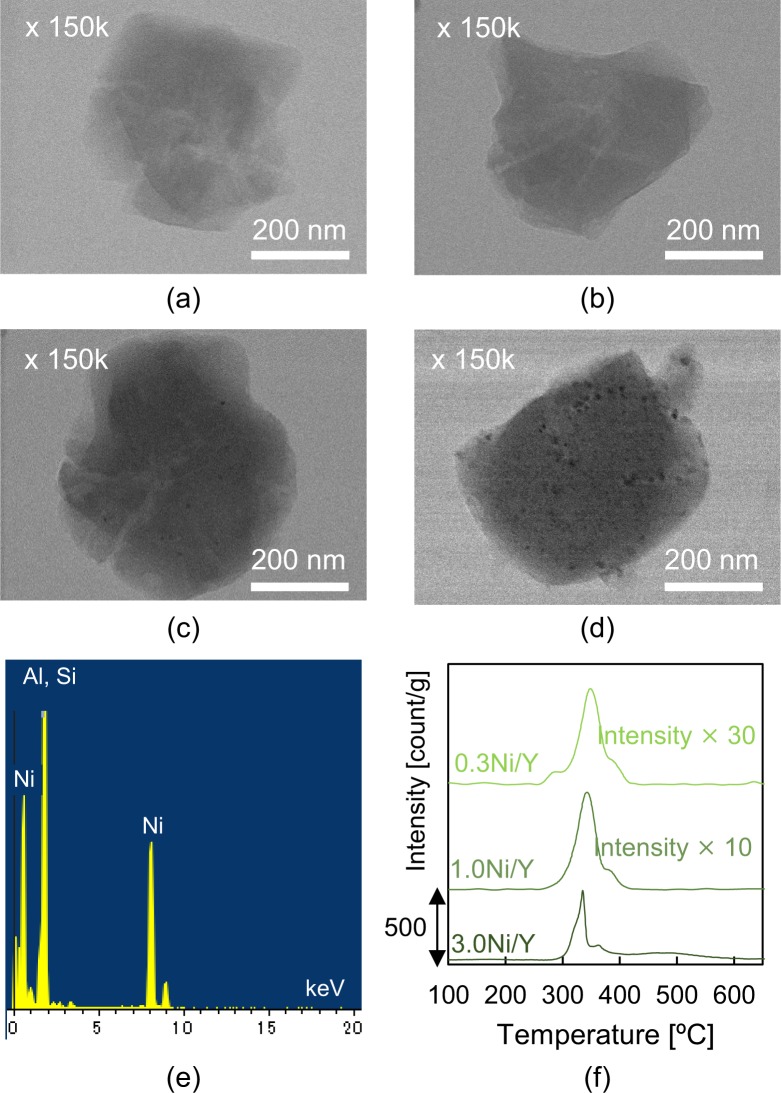
TEM images of (**a**) Y-zeolite, (**b**) 0.3Ni/Y, (**c**) 1.0Ni/Y and (**d**) 3.0 Ni/Y. (**e**) EDX spectrum of 0.3Ni/Y. (**f**) Temperature-programmed reduction (TPR) profiles of the Ni/Y catalysts.

Among all the zeolite catalysts tested, Y-zeolite showed the best catalytic ability for iPrP dealkylation and phenol production from IPP from all tested zeolite catalysts; therefore, Ni-supported Y-zeolites with different Ni loadings were synthesised. Ni was loaded without any structural damages to the zeolite frame (Fig. S2). Fig. 6 shows the TEM images of the original Y-zeolite and the Ni/Y catalysts with different amounts of Ni (0.3Ni/Y, 1.0Ni/Y, and 3.0 Ni/Y). The TEM images of the Y-zeolite and 0.3Ni/Y were comparable because the Ni particles were not observed in the 0.3Ni/Y TEM image due to the small Ni loading. Simultaneously, the energy-dispersive X-ray (EDX) spectrum of 0.3Ni/Y confirmed the presence of Ni in the catalyst (Fig. 6(e)). In contrast, the Ni particles were clearly observed in the TEM images of 1.0Ni/Y and 3.0Ni/Y, with an average particle sizes of 8 nm and 13 nm, respectively, determined by equation (1) in the SI. The TPR profiles of the synthesised catalysts (Fig. 6(f)) showed that the initial NiO reduction temperature decreased with decreasing Ni loading amount, indicating the presence of smaller Ni particles in 0.3Ni/Y than in 1.0Ni/Y and 3.0Ni/Y.

### Evaluation of the Ni/Y catalysts for the conversion of IPP into phenol via hydrogenation and dealkylation reactions

The effects of the synthesised Ni/Y catalysts on the conversion of IPP were investigated using the H_2_-TR-GC/MS system. The obtained GC/MS chromatograms, IPP conversions, and product distribution data are summarised in Fig. 4(e,f). Chromatograms obtained in the absence of a catalyst and in the presence of Y-zeolite were used for comparison. All the catalysts achieved >99% IPP conversion (Fig. 4(f)). Surprisingly, both 0.3Ni/Y and 1.0Ni/Y showed full IPP conversion and 93% phenol selectivity, much higher than those obtained by the Y-zeolite alone. This was due to the hydrogenation of IPP into iPrP and subsequent dealkylation of iPrP by the Ni/Y catalysts (Fig. 5(c)). However, the phenol selectivity decreased substantially to 40% when 3.0Ni/Y was employed, while the amounts of iPrP and hydrodeoxygenated products such as benzene and alkyl benzenes increased. The rise in the iPrP amount suggests that the dealkylation ability decreased possibly due to the Ni covering reducing the number of acid sites. Moreover, the hydrodeoxygenation was enhanced substantially by the increase in H_2_ concentration to 48 vol%, resulting in 66% selectivity for benzene and alkyl benzenes, and 22% selectivity for phenol in the liquid products. Thus, it can be concluded that higher H_2_ concentration and higher Ni loading are unsuitable for recovering phenol in this reaction system. Notably, the shift in retention time under 48 vol% H_2_ flow (Fig. 4(e)) was due to the lowered carrier gas viscosity. Thus, we confirmed that the reaction with 0.3Ni/Y under 10 vol% H_2_ achieved 100% IPP conversion and 93% phenol selectivity.

### Monitoring the deactivation of 0.3Ni/Y

**Figure 7:**
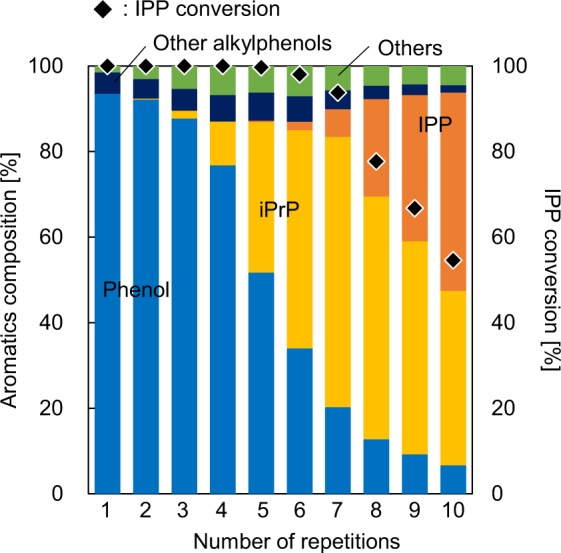
Aromatics composition and IPP conversion data obtained from the catalytic conversion of IPP using 0.3Ni/Y over 10 subsequent runs.

The conversion of IPP into phenol using the 0.3Ni/Y catalyst under 10 vol% H_2_ atmospheric flow was repeated 10 times to monitor the deterioration of the catalytic ability. The IPP conversion and liquid composition data obtained for each run are summarised in Fig. 7. The first run showed a complete IPP conversion with 94% phenol selectivity. Moreover, complete IPP conversion was observed until the end of the forth repetition and the amount of iPrP seemingly increased with every following repetition. However, the IPP conversion decreased significantly after the 7^th^ repetition, reaching 55% after 10 repetitions. The phenol selectivity in the liquid products also decreased to 7% in the 10^th^ repetition. Although optimization of the catalyst bed temperature, feed concentration, and carrier gas flow, as well as the reduction of catalyst/sample ratio, are needed for process improvement in the future, important information regarding catalyst behaviour has been provided herein using a new H_2_-TR-GC/MS system.

**Figure 8:**
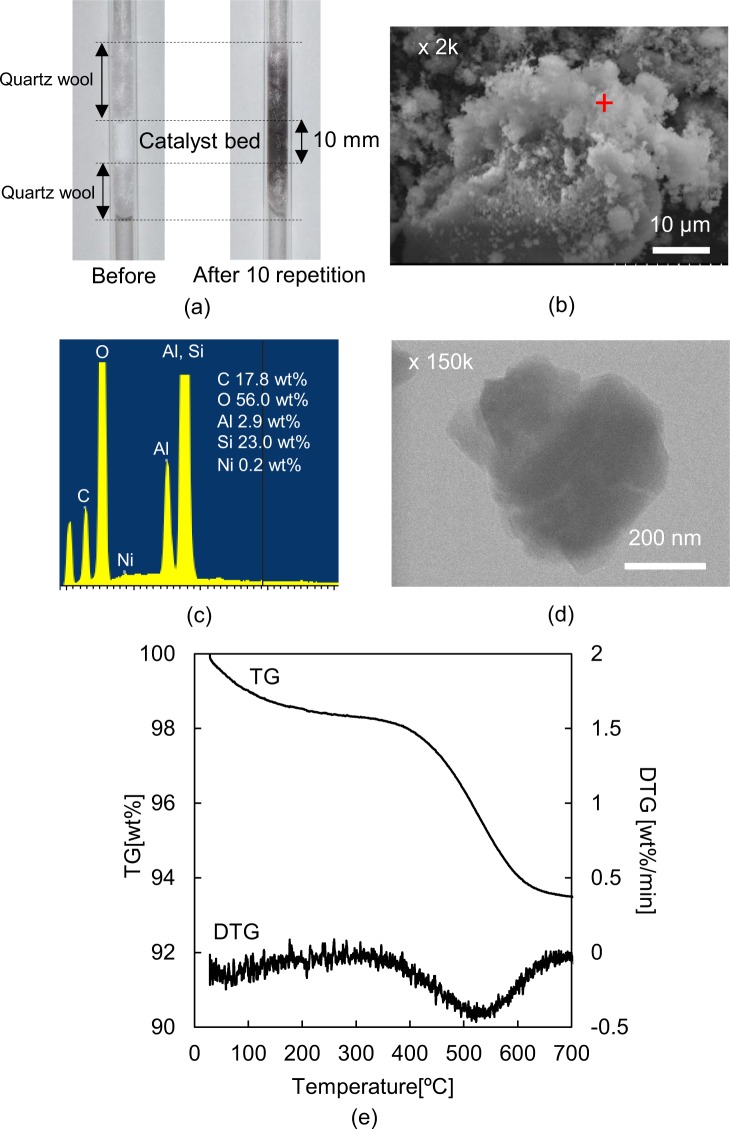
(**a**) Pictures of the catalyst bed before and after 10 subsequent repetitions. (**b**) SEM image of 0.3Ni/Y after 10 repetitions. (**c**) EDX spectrum and weight composition of the point (+) in Fig. 8(b). (**d**) TEM image of 0.3Ni/Y after 10 repetitions. (**e**) TPO thermogram of 0.3Ni/Y after 10 subsequent repetitions.

Comparison between images of the catalyst bed before and after the 10 subsequent repetitions (Fig. 8(a)) indicates that coke was deposited on the catalyst. Since coke is preferably deposited on the acid centre, the acid sites in this case were preferentially covered by coke. Moreover, the fact that the dealkylation of iPrP was deactivated at an early repetition stage suggests that the zeolite pores were blocked by coke deposition during the repetitions. This, in turn, resulted in reduced availability of the acidic sites and lowered access of IPP to the Ni-support in the pores, which is one of the reasons for the decrease in IPP conversion with every repetition. A SEM image and EDX spectrum of the used 0.3Ni/Y (Fig. 8(b–d)) confirmed the coke deposition on the catalyst. However, the type of coke was indistinguishable. Moreover, the Ni particles were still not observed even after 10 repetitions (Fig. 8(d)). When Ni is sintered, it forms bigger particles after considerable amount of time^63,64^, implying that the conditions used herein did not produce significant Ni sintering.

The temperature programmed oxidation (TPO) thermogram (Fig. 8(e)) of 0.3Ni/Y after 10 repetitions shows a two-step weight loss. The first step (until 200 °C) corresponds to the release of moisture from the catalyst^65,66^, while there are two possibilities could account for the second weight loss (starting from 400 °C): combustion of either the layered or the filamentous carbon deposited on the catalyst^67^. The fact that no filamentous carbon was detected in the SEM analysis suggests that the deposited coke is in fact layered carbon.

In this work, we employed H_2_-TR-GC/MS for the first time to investigate the possibility of selective phenol recovery via simultaneous hydrogenation/dealkylation of compounds with low industrial demand such as IPP and iPrP. Below, we have summarise the main results and conclusions from this work.

1. The Y-zeolite showed the best iPrP dealkylation ability, with >99% conversion and phenol selectivity at 350 °C. Since the pore size (0.90 nm) of the Y-zeolite was large enough to accept the iPrP molecules (0.64 nm), the dealkylation reaction was promoted on the acidic sites inside the pores. In addition, the Y-zeolite achieved IPP conversion of over 99%, while simultaneously providing lower phenol selectivity (74%) due to the polymerisation of IPP molecules and the subsequent cracking of the IPP polymers, which were simultaneously catalysed on the acid sites.
2. Complete IPP conversion and 92% phenol selectivity was obtained from the synthesised 0.3Ni/Y catalyst in the presence of 10 vol% H_2_ atmospheric flow via a hydrogenation of the isopropenyl unit of IPP on the Ni-support and subsequent dealkylation on the acid sites in the Y-zeolite. The repetitive use of the catalyst led to coke deposition and subsequent deactivation of the hydrogenation and dealkylation abilities, which were successfully monitored using an H_2_-TR-GC/MS system.
3. In this study, IPP and iPrP (low industrial demand), which are the main pyrolysates of BPA skeletal polymers, could be selectively converted into phenol (high industrial demand) using relatively low-cost Ni/Y catalysts under mild conditions. The findings presented herein can allow the recovery of phenol-rich products from BPA skeletal polymeric waste and can also be expanded to recover phenol-rich products from alkyl phenols derived from lignocellulosic materials.
4. We believe that the novel methodology proposed herein, viz. the combination of tandem micro-reactor, H_2_-generator and GC/MS, can be employed to evaluate catalytic hydrogenation and dealkylation systems, and thus contribute substantially to the fields of green chemistry and reaction engineering.

## Methods

### Materials

Reagent-grade 4-isopropyl phenol (iPrP) and 4-isopropenyl phenol (IPP) were obtained from Tokyo Chemical Industry Co., Ltd. (Tokyo, Japan) and Kanto Chemical Co., Ltd. (Tokyo, Japan), respectively. Protonic zeolites HOA822 (Z5-24), HOA840 (Z5-40), HOA890 (Z5-1500), HUA360 (Y-zeolite), HOA640 (MOR), and HOA722 (FER) with particle sizes less than 10 μm were supplied from Tosoh Corporation (Tokyo, Japan). The characteristics of the zeolites used in this work are summarised in Table 1. The acid site densities of the zeolites were determined via ammonia temperature-programmed desorption (NH_3_-TPD) measurements, the detail analytical methods for which are summarised in SI. Even though zeolites Z5-24, Z5-40 and Z5-1500 have the same size pore (0.58 nm), they exhibited very different acid site densities (1.14, 0.51 and 0.08 mmol/g, respectively). In comparison, the Y-zeolite, which has the biggest pore size (0.90 nm) among the zeolites used herein, had relatively low acid site density (0.14 mmol/g). On the other hand, the zeolite with the smallest pores (0.48 nm) among the zeolites used herein, FER, exhibited the highest acid site density (1.45 mmol/g). Additionally, MOR had an acid density (0.49 mmol/g) comparable to Z5-40, while its pores (0.70 nm) were bigger than those of the ZSM-5 zeolites.

Structure optimisation of iPrP and IPP was carried out with Gaussian R 16 W^58^, a computational chemistry software, using the density functional theory. The kinetic diameter of these molecules was calculated by the method reported by Wang and Frenklach^59^ (Fig. 3 and SI). In general, the kinetic diameter is the most important diameter to consider when examining the adaptability of molecules to the zeolite pores.

### Synthesis and characterisation of the Ni/Y catalysts

Y-zeolite with different Ni loading amounts was synthesised via the impregnation method. The desired amount of Ni nitrate hexahydrate (Ni(NO_3_)_2_·6H_2_O) (0.074, 0.246 or 0.742 g), 5 g of Y-zeolite, and 100 mL ion-exchanged water were mixed in a 300-mL glass beaker. The mixed solution was then stirred (400 rpm) at 100 °C until the water was completely evaporated. While the amount of Ni appears to be nominal, there is little doubt that the Ni in the solution was supported on the Y-zeolite, as neither the Ni or the Y-zeolite could evaporate from the beaker. The recovered solid was calcined at 200 °C for 16 h in air to obtain NiO/Y. The synthesised Ni/Y compounds with 0.3, 1.0 and 3.0 wt% Ni loadings were named 0.3Ni/Y, 1.0Ni/Y, and 3.0Ni/Y, respectively. Before all the following experiments were conducted, the NiO/Y catalysts were converted to Ni/Y in a reactor (more details available in the next section). The synthesised catalysts were then characterised via temperature-programmed reduction (TPR), X-ray diffraction (XRD), scanning electron microscopy-energy dispersive X-ray spectroscopy (SEM-EDX) and field emission-scanning transmission electron microscopy (FE-STEM). The detailed analytical conditions are summarised in the SI.

### H_2_-TR-GC/MS experiments

The TR-GC/MS system (TR: Rx-3050 TR, Frontier Laboratories Ltd. (Koriyama, Japan); GC: 7890 A, Agilent Technologies (Tokyo, Japan); Column: Ultra ALLOY® metal capillary column UA^+^-1, Frontier Laboratories Ltd. (Koriyama, Japan)) reported in our previous papers^56,57^, was combined with an H_2_ generator (HG270, H_2_ purity: over 99.99%, GL Sciences (Tokyo, Japan)) and an H_2_ mass flow controller (T1000, Fujikin, Osaka, Japan). The resulting system was abbreviated as the H_2_-TR-GC/MS system (Fig. 2). The detailed conditions are summarised in the SI.

#### iPrP and IPP conversion using different zeolites

A sample holder, filled with 0.5 mg iPrP or IPP, was placed in the upper part (outside the heating zone) of the first micro-reactor. The flow rate was set at 104 mL/min (100:1 of split ratio; column flow rate of 1 mL/min; septum purge rate of 3 mL/min). The combination of 0.5 mg sample and the split ratio 100:1 was suitable for obtaining reliable peak intensity and good peak shape. A quartz tube reactor in the second micro-reactor was charged with the catalyst (15 mg). The catalyst amount used was the minimum amount needed for the characterization of spent catalysts after the experiments. For the non-catalytic experiments, only quartz wool was filled in the second micro-reactor. The temperature of the first micro-reactor was set at 300 °C because this is adequate to rapidly evaporate both iPrP and IPP^20^. The temperature of the second micro-reactor was set to 350 °C because it has previously been reported that *n*-propylphenol is effectively dealkylated using ZSM-5 at this temperature^37^. When the conditions were stabilised, the sample holder was dropped into the heating zone of the first micro-reactor, where iPrP and IPP were rapidly vaporised and carried into the second micro-reactor. The products generated from the TR were directly introduced into GC/MS with a separation column (UA^+^-1) and analysed without a product recovery process.

#### IPP conversion using Ni/Y catalysts with different Ni loadings

The same amounts of IPP and synthesised catalysts were filled into the TR. The second micro-reactor was heated to 400 °C under 48 vol% H_2_/He flow (H_2_: 50 mL/min + He: 54 mL/min) for 90 min in order to reduce NiO/Y to Ni/Y. Then, the carrier gas was switched to 10 vol% H_2_/He flow (H_2_: 10 mL/min + He: 94 mL/min) or left uncharged, and the temperatures of the first and second micro-reactors were set at 300 and 350 °C, respectively. Then, IPP was supplied to the first micro-reactor. The remaining procedure is the same as the described in section (*i*).

#### Repeated IPP conversion using the 0.3Ni/Y catalyst

The IPP conversion using 0.3Ni/Y was repeated 10 times without a catalyst replacement. The procedures for the catalyst pretreatment, sample injection, and product analysis by GC/MS were the same as in the previous sections. Catalyst reduction between each run was avoided.

## Calculations

The conversion rate of iPrP and IPP (*C*_iPrP/IPP_ [%]), and the phenol selectivity (*S* [%]) were defined as follows:

1 $${C}_{\mathrm{iPrP}/\mathrm{IPP}}=(1-\frac{{A}_{\mathrm{iPrP}/\mathrm{IPP}}}{{A}_{{\rm{all}}}})\times 100$$

2 $$S=\frac{{A}_{{\rm{phenol}}}}{{A}_{{\rm{arom}}}}\times 100$$

where *A*_iPrP_, *A*_IPP_, *A*_phenol_, *A*_arom_, and *A*_all_ are the peak areas of the chromatograms of iPrP, IPP, phenol, all products containing aromatic rings, and all products standardized by the input sample weight, respectively.

## Electronic supplementary material

Revised supplementary information
