# Supplementary material for: Selective phenol recovery via simultaneous hydrogenation/dealkylation of isopropyl- and isopropenyl-phenols employing an H2 generator combined with tandem micro-reactor GC/MS
Source: Sci Rep. 2018 Sep 18;8:13994. doi: 10.1038/s41598-018-32269-6 (PMC6143636; doi:10.1038/s41598-018-32269-6)
Supplement: Supplementary file 1 — Revised supplementary information [file 41598_2018_32269_MOESM1_ESM.doc]

**Supplementary Information**

Selective phenol recovery via simultaneous hydrogenation/dealkylation of isopropyl- and isopropenyl-phenols employing an H_2_ generator combined with tandem micro-reactor GC/MS

Shogo Kumagai,*^a^ Masaki Asakawa,^a^ Tomohito Kameda,^a^ Yuko Saito,^a^ Atsushi Watanabe,^b^ Chuichi Watanabe,^b^ Norio Teramae ^b,c^ and Toshiaki Yoshioka ^a^

*^a^* Graduate School of Environmental Studies, Tohoku University, 6-6-07 Aoba, Aramaki-aza, Aoba-ku, Sendai, Miyagi 980-8579, Japan

*^b^* Frontier Laboratories Ltd., 4-16-20, Saikon, Koriyama, Fukushima 963-8862, Japan

*^c^* Department of Chemistry, Graduate School of Science, Tohoku University, Aoba-ku, Sendai, Miyagi 980-8578, Japan

^*^Corresponding author. Email: kumagai@tohoku.ac.jp; tel./fax: +81-22-795-7212.

*Characterization of fresh and used catalysts*

The SiO_2_/Al_2_O_3_ molar ratio [mol/mol] and pore diameter [nm] of the purchased six zeolites were provided by Tosoh Corporation. Acid site density of the purchased zeolites was investigated by ammonia temperature-programmed desorption (NH_3_-TPD) (Quantachrome Instruments, ChemBET PULSAR^TM^ TPR/TPD). NH_3_ adsorption study was performed at 120 °C for 1 h under 10 vol% NH_3_/He flow, and NH_3_ desorption was monitored under He flow over the following temperature program: 120 °C → 10 °C/min → 900 °C. All TPD profiles (Fig. S1) showed 2-step NH_3_ desorption (first step: less than ~330 °C, second step: more than ~330 °C), where only the second step was considered for acid site density calculation because the first step corresponds to desorption of NH_3_ adsorbed on NH^4+^. The measurements were controlled using TPRWin software (Quantachrome Instruments). The NH_3_ desorption areas were determined using the software QCfit (Quantachrome Instruments).

Temperature-programmed reduction (TPR) was carried out for the synthesized Ni/Y using ChemBET PULSAR^TM^ TPR/TPD. H_2_ reduction was monitored under 10 vol% H_2_/Ar flow over the following temperature program: 40 °C → 10 °C/min → 650 °C. The measurements were controlled using TPRWin software (Quantachrome Instruments). In addition, X-ray diffraction (XRD, Rigaku powder diffractometer RINT-2200VHF+/PC, scanning between 3° and 90° using the CuKα line), scanning electron microscopy-energy dispersive X-ray spectroscopy (SEM-EDX; Hitachi-High-Technologies Corporation, S-4800), and field-emission scanning transmission electron microscopy (FE-STEM, Hitachi-High-Technologies Corporation, HD-2700) were conducted. TEM observation revealed the presence of Ni particles in 1.0Ni/Y and 3.0Ni/Y. The Ni particle size was estimated by equation (**1**):

$\sum n_{i}d_{i}^{3}/\sum n_{i}d_{i}^{2}$ (**1**)

where *d*_i_ is size of Ni particle [nm] and *n_i_* is number of Ni particle with the size of *d*_i_.

The used 0.3Ni/Y catalyst (*(iii) Repeated IPP conversion using the 0.3Ni/Y catalyst*) was investigated by XRD, SEM-EDX, TEM and temperature-programmed oxidation (TPO) over the following temperature program: 40 °C → 15 °C/min → 800 °C.[^1^](#_ENREF_1)

**Fig. S1** Ammonia temperature-programmed desorption (NH_3_-TPD) profiles of zeolites used in the present work.

**Fig. S2** XRD patterns of Y-zeolite and Ni/Y catalysts.

*Structure optimization of iPrP and IPP and calculation of kinetic diameter*

Molecule geometries were optimised with the default (eigenvalue-following) optimization algorithm using B3LYP hybrid functional and 6-31+G(*d*,*p*) basis set to compute the energy. The kinetic diameter was determined by equation (**2**) reported by Wang and Frenklach[^2^](#_ENREF_2):

$\sigma=1.234\times\left(M_{\text{W}}\right)^{\frac{1}{3}}$ (**2**)

where *M*_w_ [g/mol] is the molecular weight of compounds. This kinetic diameter estimation assumes a spherical molecule; hence the critical mass is related to the size of the sphere. This equation was established for hydrocarbons. Notably, Jae et al.[^3^](#_ENREF_3) revealed that the equation is also applicable for oxygen-containing molecules.

**Fig. S3** Three-dimensional views of IPP dimers optimized by Gaussian R 16W and kinetic diameters determined by the method reported by Wang and Frenklach.[^2^](#_ENREF_2)

*H_2_-TR-GC/MS experiments*

The tandem micro-reactor GC/MS (TR-GC/MS) system (TR: Rx-3050 TR, Frontier Laboratories Ltd. (Koriyama, Japan); GC: 7890A, Agilent Technologies (Tokyo, Japan); Column: Ultra ALLOY® metal capillary column UA^+^-1, Frontier Laboratories Ltd. (Koriyama, Japan)) reported in our previous paperss was combined with an H_2_ generator (HG270, H_2_ purity: over 99.99%, GL Sciences (Tokyo, Japan)) and an H_2_ mass flow controller (T1000, Fujikin, Osaka, Japan). The resulting system was abbreviated as the H_2_-TR-GC/MS system (Fig. 2 in the main manuscript). GC/MS conditions were set as follows: column flow rate, 1 mL/min; injector split ratio, 100:1; septum purge, 3 mL/min; MSD source temperature: 230 °C; MS quadrupole temperature: 150 °C; acquisition mode, scan; scanning range, *m/z* = 10–800.

*(i) iPrP and IPP conversion using different zeolites:* A sample holder, filled with 0.5 mg iPrP or IPP, was placed inside the upper part (outside the heating zone) of the first micro-reactor. A quartz tube reactor in the second micro-reactor was charged with the catalyst (15 mg). Temperatures of the first and second micro-reactors were set at 300 and 350 °C, respectively, under He flow (104 mL/min). The temperature of 300 °C in the first micro-reactor was sufficient for rapid evaporation of both iPrP and IPP,[^6^](#_ENREF_6) and the temperature of 350 °C in the second micro-reactor was selected because Verboekend et al.[^7^](#_ENREF_7) reported that dealkylation of *n*-propylphenol using HZSM-5 was effective at 350 °C. When the conditions were stabilized, the sample holder was dropped into the heating zone of the first micro-reactor, where iPrP and IPP were rapidly vaporized and carried into the second micro-reactor. The products generated from the TR were directly introduced into a GC/MS with a separation column (UA^+^-1) and analysed without a product recovery process. The products introduced in the column were analyzed under a GC oven program (40 °C (5 min) → 10 °C min^−1^ → 300 °C (10 min)), which was initiated simultaneously with the sample holder injection. Each peak of the obtained MS spectra was compared with those in the NIST08 library. We identified each peak with >80% similarity; peaks with <80% similarity were categorized as ‘others’.

*(ii) IPP conversion using Ni/Y catalysts with different Ni loadings:* The same amounts of IPP and synthesized catalysts as employed in procedure (*i*) were filled into the TR. The second micro-reactor was heated up to 400 °C under 48 vol% H_2_/He flow (H_2_: 50 mL/min + He: 54 mL/min) for 90 min to reduce NiO/Y to Ni/Y. Then, the carrier gas was switched to 10 vol% H_2_/He flow (H_2_: 10 mL/min + He: 94 mL/min) or left uncharged, and the temperatures of the first and second micro-reactors were set to 300 and 350 °C, respectively. Then, IPP was supplied to the first micro-reactor. The remaining procedure was the same as that described in section (*i*).

*(iii) Repeated IPP conversion using the 0.3Ni/Y catalyst:* The IPP conversion using 0.3Ni/Y was repeated 10 times without replacing catalyst. The procedures for the catalyst pretreatment, sample injection, and product analysis by GC/MS were the same as in the previous sections ((*i*) and (*ii*)). Catalyst reduction between each run was avoided.

**Fig. S4** Conversion rate of iPrP or IPP vs. acid site density of Z5 zeolites.

**Fig. S5** XRD spectrum of original Y-zeolite and 0.3Ni/Y before and after 10 repetitions.

**Table S1.** Catalytic conversion products from iPrP in the presence and absence of zeolites

|  |  | No catalyst | Z5-24 | Z5-40 | Z5-1500 | Y-zeolite | MOR | FER |
| --- | --- | --- | --- | --- | --- | --- | --- | --- |
| **Gas** | | **-** | **54.4** | **19.3** | **4.3** | **33.5** | **46.8** | **27.1** |
|  | Propylene | - | 54.4 | 19.3 | 4.3 | 33.5 | 46.8 | 27.1 |
| **Liquid** | | **99.8** | **45.0** | **80.5** | **95.6** | **65.4** | **52.8** | **72.7** |
|  | Phenol | - | 43.4 | 30.7 | 12.5 | 64.7 | 52.5 | 46.6 |
|  | Isopropyl phenol (iPrP) | 99.4 | 1.6 | 49.8 | 82.8 | 0.7 | 0.2 | 26.2 |
|  | Isopropenyl phenol (IPP) | 0.4 | - | - | 0.3 | - | - | - |
|  | Methyl phenol | - | - | - | - | + | - | - |
|  | Propyl phenol | - | - | - | - | + | - | - |
| **Others** | | **0.2** | **0.6** | **0.2** | **+** | **1.1** | **0.5** | **0.1** |
| Total/area% | | 100.0 | 100.0 | 100.0 | 100.0 | 100.0 | 100.0 | 100.0 |
| -: Not detected, +: < 0.1 | |  |  |  |  |  |  |  |

**Table S2.** Catalytic conversion products from IPP in the presence and absence of zeolites

|  |  | No catalyst | Z5-24 | Z5-40 | Z5-1500 | Y-zeolite | MOR | FER |
| --- | --- | --- | --- | --- | --- | --- | --- | --- |
| **Gas** | | **-** | **6.5** | **-** | **-** | **14.6** | **23.8** | **5.7** |
|  | Propylene | - | 6.5 | - | - | 14.6 | 23.8 | 5.7 |
| **Liquid** | | **98.9** | **91.8** | **98.9** | **100.0** | **85.0** | **76.2** | **92.4** |
|  | Phenol | 0.2 | 45.6 | 32.1 | 3.5 | 62.9 | 44.5 | 40.5 |
|  | Isopropyl phenol (iPrP) | 0.8 | 0.7 | - | 0.3 | 11.7 | 3.7 | 24.6 |
|  | Isopropenyl phenol (IPP) | 91.4 | 38.3 | 52.0 | 96.2 | 0.7 | 24.1 | 24.8 |
|  | Methyl phenol | - | 6.2 | 2.3 | - | 2.9 | 0.6 | 0.7 |
|  | Ethyl phenol | - | 1.0 | - | - | 6.1 | 2.0 | 1.5 |
|  | Propyl phenol | - | - | - | - | 0.3 | - | 0.2 |
|  | Other alkyl phenols | 0.3 | - | 0.7 | - | - | - | - |
|  | Benzofurans | + | - | 3.2 | - | - | - | - |
|  | Naphthalenes | - | - | 8.7 | - | 0.4 | 1.3 | - |
|  | IPP dimers | 6.2 | - | - | - | - | - | - |
| **Others** | | **1.1** | **1.7** | **1.1** | **0.0** | **0.4** | **0.0** | **1.9** |
| Total/area% | | 100.0 | 100.0 | 100.0 | 100.0 | 100.0 | 100.0 | 100.0 |
| -: Not detected | |  |  |  |  |  |  |  |

**Table S3.** Catalytic conversion products from IPP in the presence and absence of Ni loaded Y-zeolite

|  |  | No catalyst | Y-zeolite | 0.3Ni/Y (10vol% H_2_) | 1.0Ni/Y (10vol% H_2_) | 3.0Ni/Y (10vol% H_2_) | 3.0Ni/Y (48 vol% H_2_) |
| --- | --- | --- | --- | --- | --- | --- | --- |
| **Gas** | | **-** | **14.6** | **16.1** | **10.3** | **6.2** | **29.5** |
|  | Methane | *-* | *-* | *-* | *-* | *-* | *12.2* |
|  | Propylene | - | 14.6 | 16.1 | 10.3 | 6.2 | 17.3 |
| **Liquid** | | **98.6** | **85.0** | **82.9** | **88.5** | **93.4** | **70.5** |
|  | Phenol | 0.2 | 62.9 | 76.2 | 81.9 | 36.8 | 15.6 |
|  | Isopropyl phenol (iPrP) | 0.8 | 11.7 | 1.9 | 2.0 | 27.5 | 2.9 |
|  | Isopropenyl phenol (IPP) | 91.4 | 0.7 | - | - | 1.1 | - |
|  | Methyl phenol | - | 2.9 | 1.4 | 1.2 | 2.6 | 3.0 |
|  | Ethyl phenol | - | 6.1 | 1.3 | 1.4 | 2.3 | - |
|  | Propyl phenol | - | 0.3 | - | - | - | 0.1 |
|  | Benzene | - | - | - | - | 8.8 | 28.1 |
|  | Toluenes | - | - | - | - | 2.6 | 11.0 |
|  | Ethyl benzene | - | - | - | - | 0.5 | 2.6 |
|  | Isopropyl benzene | - | - | - | - | 1.6 | 3.3 |
|  | Xylene | - | - | - | - | 0.5 | 1.5 |
|  | Indenes | - | - | - | - | 1.5 | 0.5 |
|  | Benzofurans | - | - | 1.5 | 2.1 | 0.2 | - |
|  | Naphthalenes | - | 0.4 | 0.6 | - | 7.3 | 1.9 |
|  | IPP dimers | 6.2 | - | - | - | - | - |
| **Others** | | **1.4** | **0.4** | **1.0** | **1.2** | **0.4** | **-** |
| Total/area% | | 100.0 | 100.0 | 100.0 | 100.0 | 100.0 | 100.0 |
| -: Not detected | |  |  |  |  |  |  |

**Table S4.** Products distribution during repeated IPP conversion using the 0.3Ni/Y catalyst

|  |  | Repetition No. | | | | | | | | | |
| --- | --- | --- | --- | --- | --- | --- | --- | --- | --- | --- | --- |
|  |  | 1 | 2 | 3 | 4 | 5 | 6 | 7 | 8 | 9 | 10 |
| **Gas** | | **1.8** | **-** | **-** | **-** | **-** | **-** | **-** | **-** | **-** | **-** |
|  | Propylene | 1.8 | - | - | - | - | - | - | - | - | - |
| **Liquid** | | **98.2** | **100.0** | **100.0** | **99.9** | **98.5** | **98.0** | **96.5** | **97.8** | **97.2** | **97.8** |
|  | Phenol | 91.8 | 92.2 | 87.7 | 76.7 | 51.0 | 33.3 | 19.5 | 12.4 | 8.9 | 6.5 |
|  | Isopropyl phenol (iPrP) | - | 0.2 | 1.8 | 10.3 | 34.7 | 50.0 | 61.1 | 55.6 | 48.4 | 39.8 |
|  | Isopropenyl phenol (IPP) | - | - | - | - | 0.3 | 2.0 | 6.2 | 22.3 | 33.3 | 45.4 |
|  | Methyl phenol | 4.4 | 3.6 | 2.9 | 2.0 | 1.0 | 0.5 | 0.3 | 0.1 | + | + |
|  | Ethyl phenol | 0.5 | 0.9 | 2.3 | 3.8 | 5.0 | 4.8 | 3.7 | 2.7 | 2.2 | 1.6 |
|  | Propyl phenol | - | - | - | 0.4 | 0.5 | 0.5 | 0.3 | 0.2 | 0.2 | 0.1 |
|  | Isopropyl benzene | - | - | 0.7 | 2.1 | 1.8 | 2.8 | 2.1 | 1.2 | 0.9 | 0.6 |
|  | Indenes | - | - | - | 0.2 | 0.2 | 0.2 | 0.2 | 0.2 | + | + |
|  | Benzofurans | 0.8 | 1.3 | 1.3 | 1.0 | 0.7 | 0.4 | 0.2 | 0.2 | 0.2 | 0.1 |
|  | Naphthalenes | 0.7 | 1.8 | 3.2 | 3.4 | 3.4 | 3.4 | 3.0 | 2.9 | 3.0 | 3.5 |
| **Others** | | **-** | **-** | **-** | **0.1** | **1.5** | **2.0** | **3.5** | **2.2** | **2.8** | **2.2** |
| Total/area% | | 100.0 | 100.0 | 100.0 | 100.0 | 100.0 | 100.0 | 100.0 | 100.0 | 100.0 | 100.0 |
| -: Not detected, +: < 0.1 | |  |  |  |  |  |  |  |  |  |  |

**References**

1 Kumagai, S. *et al.* Novel Ni–Mg–Al–Ca catalyst for enhanced hydrogen production for the pyrolysis–gasification of a biomass/plastic mixture. *Journal of Analytical and Applied Pyrolysis* **113**, 15-21 (2015).

2 Wang, H. & Frenklach, M. Transport properties of polycyclic aromatic hydrocarbons for flame modeling. *Combustion and Flame* **96**, 163-170 (1994).

3 Jae, J. *et al.* Investigation into the shape selectivity of zeolite catalysts for biomass conversion. *Journal of Catalysis* **279**, 257-268 (2011).

4 Kumagai, S. *et al.* Tandem μ-reactor-GC/MS for online monitoring of aromatic hydrocarbon production via CaO-catalysed PET pyrolysis. *Reaction Chemistry & Engineering* **2**, 776-784 (2017).

5 Kumagai, S. *et al.* Aromatic hydrocarbon selectivity as a function of CaO basicity and aging during CaO-catalyzed PET pyrolysis using tandem µ-reactor-GC/MS. *Chemical Engineering Journal* **332**, 169-173 (2018).

6 Kumagai, S., Ono, S., Yokoyama, S., Kameda, T. & Yoshioka, T. Fate of bisphenol A pyrolysates at low pyrolytic temperatures. *Journal of Analytical and Applied Pyrolysis* **125**, 193-200 (2017).

7 Verboekend, D., Liao, Y., Schutyser, W. & Sels, B. F. Alkylphenols to phenol and olefins by zeolite catalysis: a pathway to valorize raw and fossilized lignocellulose. *Green Chemistry* **18**, 297-306 (2016).
